# The Effect of 1-Ethyl-3-Methylimidazolium Chloride on Oxidative Stress and the Functioning of the Photosynthetic Apparatus in Maize Seedlings—The Modulatory Role of Exogenous Ascorbic Acid

**DOI:** 10.3390/toxics14070589

**Published:** 2026-07-03

**Authors:** Barbara Pawłowska, Aleksandra Lechowska, Radomír Ščurek, Robert Biczak

**Affiliations:** 1 The Faculty of Science and Technology, Jan Długosz University in Czestochowa, 13/15 Armii Krajowej Av., 42-200 Czestochowa, Poland; ola.lechowska04@gmail.com (A.L.); r.biczak@ujd.edu.pl (R.B.); 2 Department of Security Services, Faculty of Safety Engineering, VSB—Technical University of Ostrava, Silesia, 700 30 Ostrava, Czech Republic; radomir.scurek@vsb.cz

**Keywords:** phytotoxicity, oxidative stress, chlorophyll fluorescence, maize, 1-ethyl-3-methylimidazolium chloride

## Abstract

Ionic liquids (ILs) are widely used chemical compounds that may pose potential risks to the environment. In the present study, the effects of 1-ethyl-3-methylimidazolium chloride (EMIMCl) on growth, photosynthetic performance, and oxidative stress in maize (*Zea mays* L.) seedlings were evaluated, and the role of exogenous L-ascorbic acid (AsA) in modulating plant responses to this stress was investigated. Plants were cultivated in soil contaminated with EMIMCl at concentrations ranging from 1 to 1000 mg·kg^−1^ of soil dry weight (DW) and treated with AsA at concentrations of 0.5–2 mM. EMIMCl significantly inhibited plant growth, reduced photosynthetic pigment content, and impaired chlorophyll fluorescence parameters, accompanied by increased hydrogen peroxide (H_2_O_2_) and malondialdehyde equivalents (MDA) levels, indicating the induction of oxidative stress. Moderate doses of AsA partially alleviated EMIMCl-induced toxicity, whereas higher AsA concentrations under severe EMIMCl contamination intensified stress symptoms. These findings demonstrate a dose-dependent and biphasic role of AsA in maize responses to EMIMCl-induced stress.

## 1. Introduction

The rapid advancement of chemical technologies over recent decades has resulted in a substantial increase in both the production and application of ionic liquids (ILs). Ionic liquids constitute a vast and structurally diverse class of compounds. Owing to their distinctive physicochemical properties, these substances have been widely implemented across numerous industrial sectors. In particular, imidazolium-based ionic liquids, including 1-ethyl-3-methylimidazolium chloride (EMIMCl), have found applications in chemical synthesis, lignocellulosic biomass processing, and electrochemical technologies [1,2,3,4]. 

Market forecasts indicate that the global ionic liquids market is expected to reach USD 7266.2 million by 2030 [5]. The continuous expansion of IL production and their growing range of applications across various industries raise concerns regarding their potential environmental impact. An increasing body of literature suggests that, despite their advantageous technological properties, many ionic liquids exhibit significant biological activity, which may pose risks to living organisms following their release into the environment. Scientific investigations have confirmed that ionic liquids have already entered environmental compartments and have been detected in river waters, among others. Due to their water solubility and limited susceptibility to biodegradation, imidazolium-based ILs may accumulate in soils and surface waters, thereby increasing the exposure risk for terrestrial organisms, including crop plants. Numerous studies report that ionic liquids can exert toxic effects on bacteria, fungi, algae, crustaceans, fish, and plants [6,7,8,9]. Previous research has demonstrated that ILs, including EMIMCl, may inhibit seed germination, impair plant growth and development, and disrupt metabolic processes in various plant species, including agriculturally important crops. Moreover, ionic liquids may display strong binding affinity to human blood proteins, which could contribute to their bioaccumulation in the human body [6,10,11,12]. 

Despite numerous studies conducted to date, the precise mechanisms underlying the phytotoxic effects of ionic liquids have not yet been fully elucidated. Growing evidence suggests that disruption of biological membrane integrity and the induction of oxidative stress play central roles in their mode of action. Imidazolium cations are capable of interacting with membrane phospholipids as well as thiol-containing proteins, which may result in destabilization of cellular structures and impaired functioning of mitochondria and chloroplasts. These disturbances can lead to excessive generation of reactive oxygen species (ROS), triggering lipid peroxidation and damage to the photosynthetic apparatus [13,14,15,16]. 

Plants have evolved complex defense strategies to maintain redox homeostasis under environmental stress conditions. A key component of these protective systems is the antioxidant network, which includes both enzymatic antioxidants and low-molecular-weight reducing compounds. Ascorbic acid (AsA) plays a particularly important role within this system by directly scavenging ROS and regenerating other antioxidants through the ascorbate–glutathione cycle. In addition, AsA functions as a cofactor for certain oxidases and participates in the biosynthesis of several phytohormones, including abscisic acid (ABA) and ethylene. It also contributes significantly to maintaining intracellular and extracellular redox balance in plants. Through these functions, AsA enables plants to respond rapidly and effectively to environmental fluctuations, thereby enhancing their tolerance to various abiotic stresses [17,18,19,20]. Numerous studies have demonstrated that exogenous application of AsA may exert beneficial effects on plants exposed to drought, high salinity, or heavy metal stress [20,21,22]. 

It should be noted, however, that the effects of AsA are not invariably protective. Under conditions of severely disrupted redox balance, or when applied at excessive concentrations, ascorbic acid may exhibit pro-oxidant properties. In particular, through participation in redox reactions—especially in the presence of transition metal ions—AsA can promote the overproduction of reactive oxygen species (ROS) [19,23,24]. This phenomenon indicates that the effectiveness of exogenous antioxidants is strongly dependent on their concentration and on the intensity of environmental stress.

Despite the increasing number of studies addressing the toxicity of ionic liquids, comprehensive analyses integrating their simultaneous effects on plant growth, photosynthetic performance, and oxidative stress—along with the potential modulation of these responses by exogenous antioxidants—remain limited. In particular, there is a scarcity of data concerning the impact of EMIMCl on economically important crop species such as maize and the potential implications for agroecosystems. Maize is the third most important cereal crop worldwide, following rice and wheat. Compared with these two cereals, however, maize has a broader range of applications. In addition to its role in human nutrition, it serves as a major component of livestock feed and is widely used in industrial and bioenergy production systems [20,25]. 

Based on the available literature data, it was hypothesized that the exposure of maize seedlings to 1-ethyl-3-methylimidazolium chloride (EMIMCl) leads to dose-dependent disturbances in growth, impairment of photosynthetic apparatus functioning, and increased oxidative stress. Furthermore, it was assumed that exogenous application of L-ascorbic acid may modulate the plant response by reducing oxidative damage. It was also hypothesized that the protective effect of AsA would not be linear and could depend on both the applied AsA concentration and the intensity of EMIMCl-induced stress, potentially resulting, under severe stress conditions, in a reduction in the protective effect or the occurrence of a pro-oxidative effect.

Accordingly, the aim of this study was to evaluate the effects of 1-ethyl-3-methylimidazolium chloride (EMIMCl) on early growth and development, photosynthetic efficiency, and oxidative stress parameters in maize seedlings. The tests were conducted at EMIMCl concentrations ranging from 0 to 1000 mg ∙ kg^−1^ of soil DW, as recommended by the OECD/OCDE [26] guidelines for this type of study. It is considered that if a plant does not react to a given compound applied at a concentration of 1000 mg ∙ kg^−1^ of soil DW, it is non-toxic to that plant. Furthermore, the potential role of exogenously applied ascorbic acid in modulating plant responses to this chemical stressor was investigated. Particular emphasis was placed on dose–response relationships and on the possibility of biphasic effects of AsA under progressively increasing EMIMCl-induced stress. To the best of our knowledge, this is the first study to examine the influence of ascorbic acid on the phytotoxicity of ionic liquids.

## 2. Materials and Methods

### 2.1. Chemicals

1-Ethyl-3-methylimidazolium chloride (EMIMCl; ≥95% purity) was obtained from Sigma-Aldrich Chemical Co. (Darmstadt, Germany) L-ascorbic acid (analytical grade) was purchased from Chempur (Piekary Śląskie, Poland). All other chemicals and reagents used for the respective analyses were of at least analytical reagent grade (≥AR grade).

### 2.2. Experimental Design

**Figure 1:**
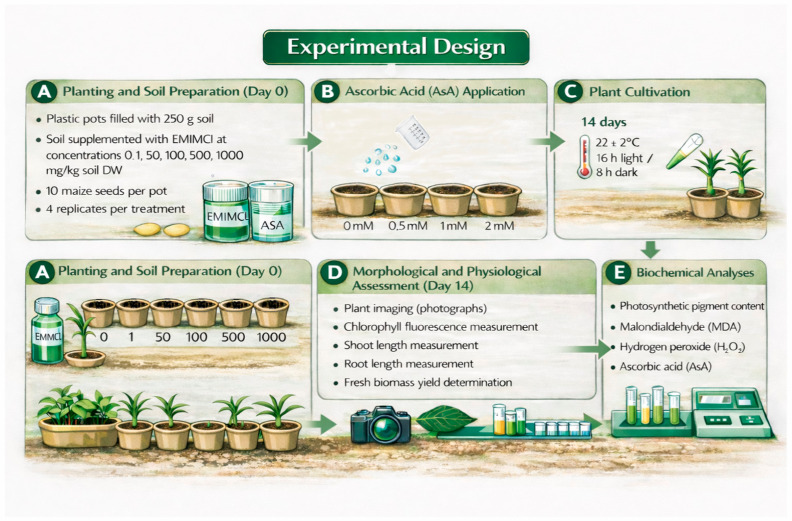
Experimental scheme for assessing EMIMCl phytotoxicity in maize under controlled conditions.

Phytotoxicity assays were conducted under controlled greenhouse conditions. The study was performed in accordance with the guidelines outlined in OECD/OCDE [26]. Ten uniform maize (*Zea mays* L.) seeds of the cultivar ‘Rywal’ were sown in plastic pots containing 250 g of control soil (without ionic liquids) or soil amended with EMIMCl at concentrations of 1, 50, 100, 500, and 1000 mg·kg^−1^ of soil dry weight (DW). Maize seeds were obtained from the Plant Breeding and Production Station in Nieznanice, belonging to Małopolska Plant Breeding—HBP Sp. z o.o. (Kobierzyce, Poland) The soil used in the experiment was classified as sandy loam, containing approximately 11% particles < 0.02 mm, 8.5 g·kg^−1^ organic carbon, and a pH (KCl) of 6.0. EMIMCl was applied to the soil in the form of aqueous solutions and thoroughly homogenized prior to sowing. Immediately after seed sowing, the soil was irrigated either with distilled water (control treatment) or with L-ascorbic acid solutions at concentrations of 0.5, 1, and 2 mM, applying 25 cm^3^ per pot. Throughout the experiment, growth conditions were maintained at constant levels: soil moisture was kept at 70% of water-holding capacity, temperature at 20 ± 2 °C, and light intensity at 170 µmol·m^−2^·s^−1^ under a 16 h light/8 h dark photoperiod. All measurements were performed in four independent replicates (Figure 1).

After 14 days of growth, chlorophyll fluorescence was measured and plant morphology was visually assessed and documented using digital photography (photos taken with a Sony DSC-H50 camera, Warsaw, Poland). Subsequently, the following parameters were determined: shoot and root length (using Image Tool, version 3.0; San Antonio, Texas, United States), fresh biomass yield, dry weight content, photosynthetic pigment concentration, and levels of H_2_O_2_, malondialdehyde (MDA), and ascorbic acid (AsA).

### 2.3. Determination of Basic Phytotoxicity Parameters

Inhibition of shoot and root growth was evaluated according to the procedure described by Wang et al. [27]. Five plants (including both shoots and roots) were selected from each pot and measured. Shoot length was determined from the base of the stem to the tip of the longest leaf, while root length was measured from the root–shoot junction to the tip of the longest root. Fresh biomass yield was also determined for each treatment.

Dry weight content was assessed following the method of Kowalska [28]. Approximately 1 g of leaf tissue was dried at 105 °C until a constant weight was achieved.

### 2.4. Chlorophyll Fluorescence

Chlorophyll fluorescence parameters were measured using an OS1p chlorophyll fluorometer (Opti-Sciences, Inc., Hudson, NH, USA). The following parameters were recorded: initial (minimal) fluorescence (F_0_), variable fluorescence (F_v_), maximal fluorescence (F_m_), the maximum quantum efficiency of photosystem II (F_v_/F_m_), and the more sensitive ratio F_v_/F_0_.

### 2.5. Determination of Photosynthetic Pigments

The concentrations of photosynthetic pigments, including chlorophyll a, chlorophyll b, total chlorophyll, and carotenoids, were determined according to the method proposed by Oren et al. [29]. Briefly, 200 mg of maize leaf tissue was homogenized in 80% acetone (4 °C) and subsequently centrifuged. Pigment concentrations were quantified spectrophotometrically by measuring absorbance at 470, 647, and 664 nm, followed by calculation using the appropriate equations.

### 2.6. Determination of Malondialdehyde Equivalents (MDA), Hydrogen Peroxide (H_2_O_2_), and Ascorbate (AsA)

Fresh plant material (0.5 g) was homogenized in 0.1% (*w*/*v*) trichloroacetic acid (TCA) and centrifuged. The resulting supernatant was used for the determination of MDA and H_2_O_2_ concentrations.

MDA equivalents assay. The reaction mixture consisted of the supernatant, 0.5% thiobarbituric acid (TBA) prepared in 20% TCA, and phosphate buffer (pH 7.6). Samples were incubated in a water bath at 95 °C for 30 minutes and then rapidly cooled in an ice bath. After cooling, absorbance was measured at 532 and 600 nm, following the method described by Hodges et al. [30]. Since the TBA assay is not fully specific for MDA but rather determines the pool of TBARS (thiobarbituric acid reactive substances), in which MDA is the major component, the results are expressed as MDA equivalents and treated as an indirect indicator of lipid peroxidation.

H_2_O_2_ assay: For hydrogen peroxide determination, the supernatant was mixed with 1 M potassium iodide (KI) and phosphate buffer (pH 7.6). The reaction mixture was incubated in the dark for 1 hour, after which absorbance was recorded at 390 nm according to Singh et al. [31].

AsA assay: To determine ascorbate content, 0.5 g of fresh maize leaf tissue was homogenized in 10% TCA and centrifuged. An aliquot of 0.2 cm^3^ of the supernatant was combined with 2% TCA, 1 M H_3_PO_4_, 0.8% bipyridyl, and 0.15% FeCl_3_. The prepared reaction mixture was incubated at 37 °C for 60 minutes in darkness. Absorbance was subsequently measured at 525 nm, and AsA concentration was calculated using a standard calibration curve, as described by Law et al. [32].

### 2.7. Statistical Analysis

The data were analyzed using two-way analysis of variance (ANOVA) to determine the effects of IL concentration, AsA concentration, and their interaction, followed by Tukey’s post hoc test (*p* < 0.05), using STATISTICA 13.3 software. Differences were considered statistically significant at *p* < 0.05. Results are presented as mean values ± standard deviation (SD). In the tables, means denoted by the same letter are not significantly different; in the figures, statistically significant differences are marked with asterisks for clarity.

Principal component analysis (PCA) was performed using standardized data (z-score normalization). The analysis was based on the correlation matrix, and the first two principal components were used for graphical interpretation. PCA calculations and visualizations were carried out in the R statistical environment (version 4.4.2) (R Foundation for Statistical Computing, Vienna, Austria).

## 3. Results

The statistical significance of the main effects of IL concentration, AsA concentration, and their interaction (IL × AsA) for all analyzed parameters was evaluated using two-way ANOVA. A complete summary of the ANOVA results (F and p values) is provided in Supplementary Table S1.

### 3.1. Phytotoxicity

The effect of different concentrations of 1-ethyl-3-methylimidazolium chloride (EMIMCl) and L-ascorbic acid (AsA) on maize emergence is presented in Table 1. The presence of EMIMCl in the soil reduced seedling emergence only at the highest tested concentration (1000 mg·kg^−1^ of soil DW). In contrast, soil supplementation with AsA at all applied concentrations mitigated the adverse effect of the ionic liquid on seed germination capacity. Even at the highest EMIMCl level, exogenous AsA application restored emergence to values that did not differ significantly from the control treatment.

Application of EMIMCl resulted in a clear, concentration-dependent inhibition of maize seedling growth, affecting both shoots and roots. The presence of AsA in the soil promoted faster shoot and root development compared with plants grown without AsA supplementation. However, AsA did not fully prevent the negative effects of the ionic liquid. Moreover, the addition of 2 mM AsA to soil containing 1000 mg·kg^−1^ of soil DW EMIMCl slightly intensified growth inhibition of both shoots and roots (Table 1).

**Figure 2:**
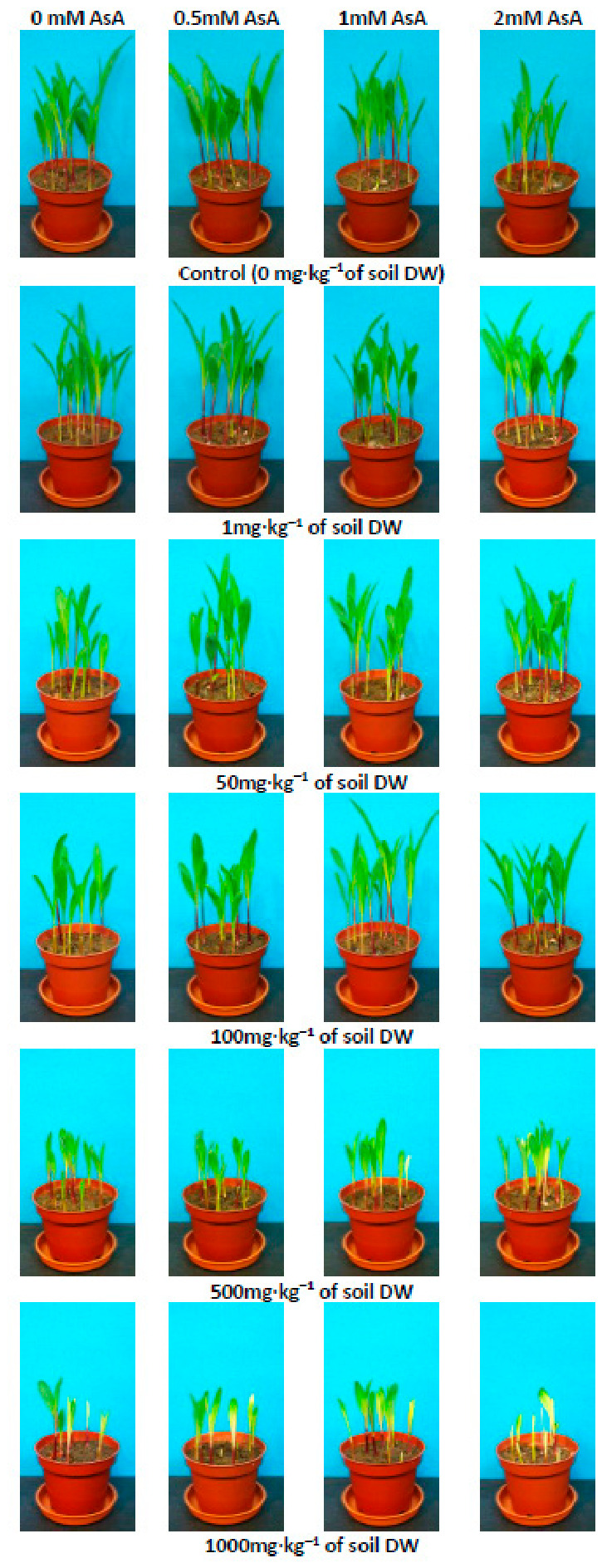
Maize seedlings (14 days after sowing) grown in soil containing different concentrations of EMIMCl and AsA.

**Figure 3:**
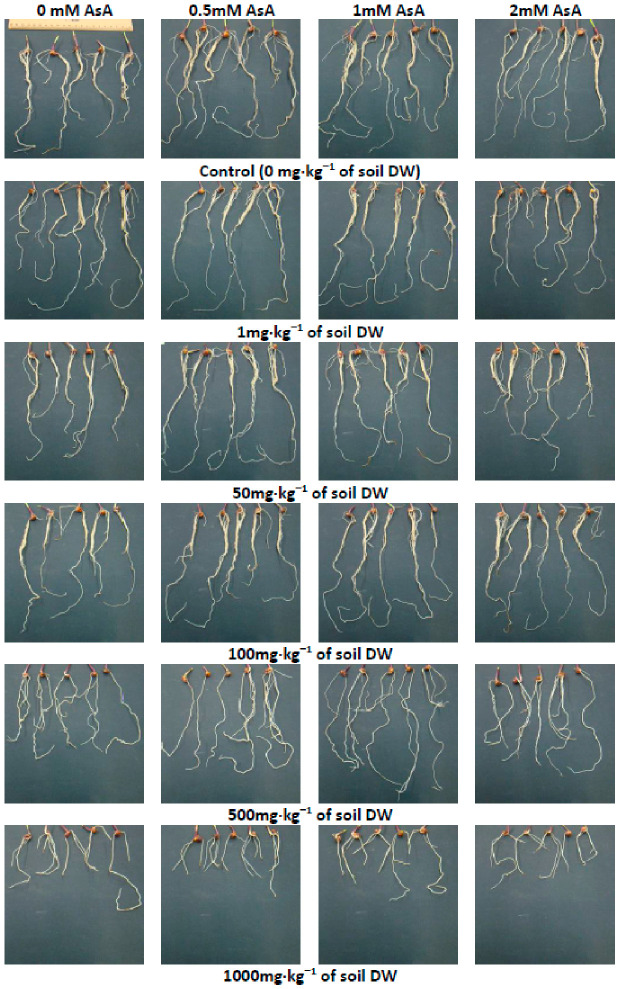
Maize root systems (14 days after sowing) grown in soil containing different concentrations of EMIMCl and AsA.

Proper germination and seedling establishment strongly influenced subsequent plant morphology and root system development (Figure 2 and Figure 3). The photographic documentation shows that EMIMCl inhibited plant and root growth in a dose-dependent manner. Furthermore, exposure to 500 and 1000 mg·kg^−1^ of soil DW EMIMCl induced chlorotic symptoms in maize leaves. When AsA was applied to soils containing the highest EMIMCl concentrations, chlorosis was more pronounced compared with plants exposed to EMIMCl alone.

These findings were reflected in fresh biomass production. The presence of EMIMCl at 1–50 mg·kg^−1^ of soil DW did not significantly affect maize fresh weight. Higher concentrations led to progressively stronger biomass reduction. In the control soil, supplementation with 0.5 and 1 mM AsA visibly increased fresh biomass yield. Similarly, in EMIMCl-contaminated soil, AsA application improved fresh weight compared with plants grown in the presence of EMIMCl alone. Importantly, following AsA addition, a significant reduction in fresh biomass was observed only from 500 mg·kg^−1^ of soil DW EMIMCl onwards. The extent of AsA-mediated mitigation of EMIMCl toxicity depended on the applied AsA concentration. Under the highest EMIMCl level (1000 mg·kg^−1^ of soil DW), a beneficial effect on fresh biomass was observed exclusively at 1 mM AsA (Table 2).

The presence of the ionic liquid also affected dry weight accumulation in maize seedlings. Exposure to 500 and 1000 mg·kg^−1^ of soil DW EMIMCl significantly increased dry weight content. The addition of AsA did not significantly alter dry weight levels compared with plants exposed to EMIMCl without antioxidant supplementation (Table 2).

### 3.2. Photosynthetic Pigment Content and Chlorophyll Fluorescence

**Figure 4:**
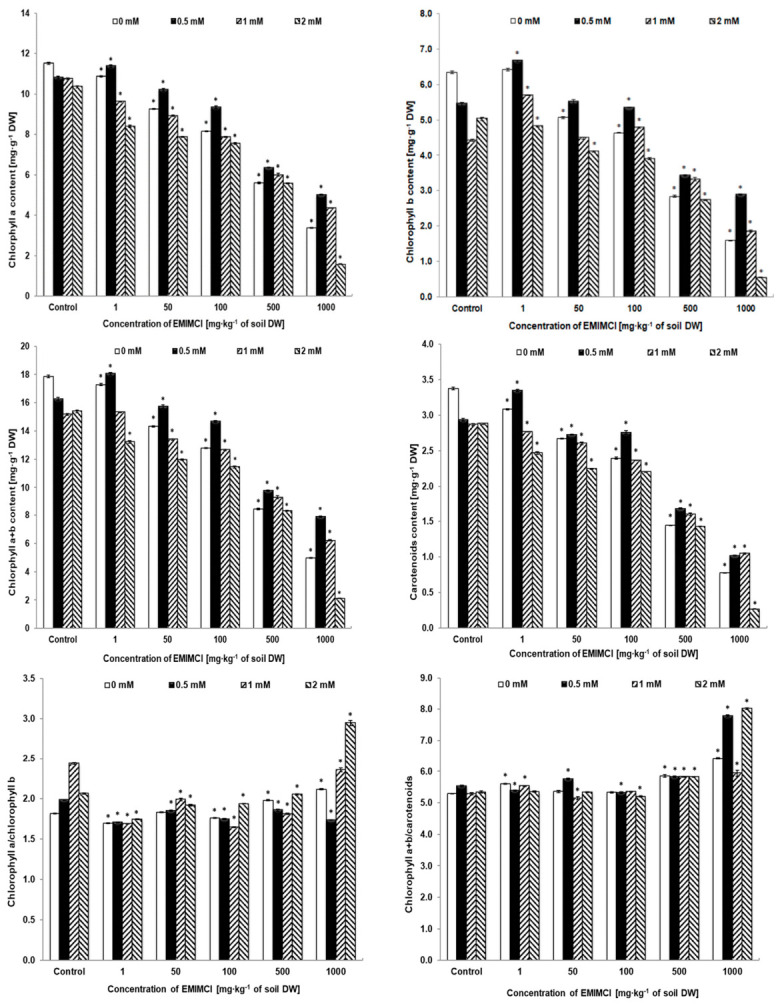
Photosynthetic pigment content in maize seedlings grown in soil contaminated with different concentrations of EMIMCl and supplemented with AsA at 0.5, 1, and 2 mM. Values represent means of four replicates (n = 4) ± standard deviation. Asterisks indicate significant differences compared with the respective control at the same AsA concentration (*p* < 0.05).

The concentrations of photosynthetic pigments (chlorophyll a, chlorophyll b, total chlorophyll, and carotenoids), as well as the ratios of chlorophyll a to chlorophyll b (chl a/chl b) and total chlorophyll to carotenoids ((chl a + chl b)/car), are presented in Figure 4.

Exposure of maize seedlings to EMIMCl resulted in a significant reduction in all analyzed photosynthetic pigments. The magnitude of this decline increased with rising concentrations of the ionic liquid. In soil not contaminated with EMIMCl, AsA supplementation led to a slight decrease in pigment content. However, when 0.5 mM AsA was applied to soil containing 1–100 mg·kg^−1^ of soil DW EMIMCl, pigment levels were higher than in plants grown without antioxidant supplementation. At higher EMIMCl concentrations (500 and 1000 mg·kg^−1^ of soil DW), beneficial effects on pigment accumulation were observed following the application of 0.5 and 1 mM AsA. In contrast, 2 mM AsA consistently reduced pigment content relative to plants grown without AsA. When high EMIMCl levels were combined with 2 mM AsA, an increase in the chl a/chl b ratio and in the total chlorophyll-to-carotenoid ratio was observed.

Chlorophyll fluorescence parameters were also evaluated, and the results are summarized in Table 3.

The presence of EMIMCl at 500 and 1000 mg·kg^−1^ of soil DW significantly increased minimal fluorescence (F_0_) and maximal fluorescence (F_m_). Simultaneously, the ratios F_v_/F_m_ and F_v_/F_0_ were reduced, indicating impairment of photosystem II efficiency. In soils containing 1–500 mg·kg^−1^ of soil DW EMIMCl, AsA supplementation did not significantly affect fluorescence parameters. Marked disturbances were observed only when AsA was applied to soil containing 1000 mg·kg^−1^ of soil DW EMIMCl. Under these conditions, 1 and 2 mM AsA nearly doubled F_0_ values, caused approximately a two-fold decrease in variable fluorescence (F_v_) and in the F_v_/F_m_ ratio, and led to a several-fold reduction in F_v_/F_0_. Moreover, the addition of 2 mM AsA to soil with 1000 mg·kg^−1^ of soil DW EMIMCl significantly decreased maximal fluorescence (F_m_).

### 3.3. MDA, H_2_O_2_, and AsA

**Figure 5:**
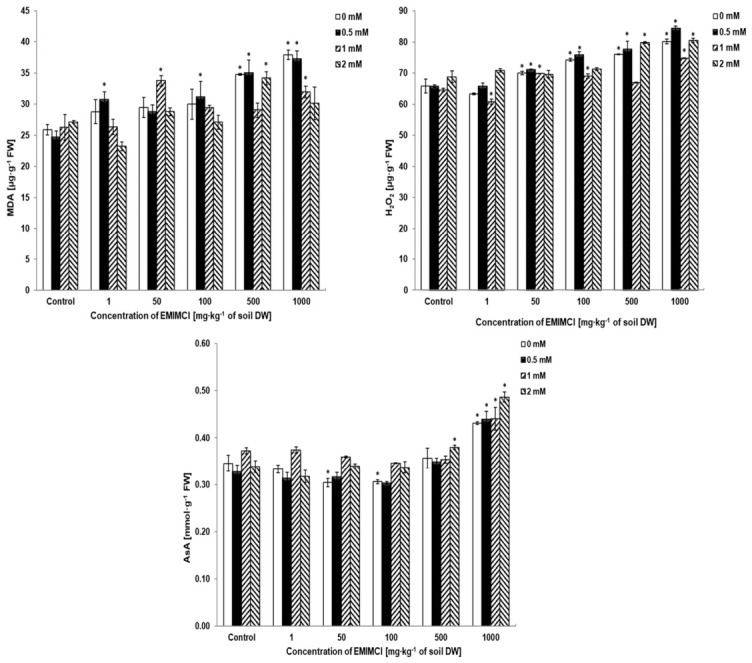
Concentrations of MDA equivalents, H_2_O_2_, and AsA in maize seedlings cultivated in soil contaminated with various levels of EMIMCl and supplemented with AsA at 0.5, 1, and 2 mM. Data are expressed as means of four replicates (n = 4) ± standard deviation. Asterisks indicate significant differences compared with the respective control at the same AsA concentration (*p* < 0.05).

The levels of H_2_O_2_, MDA equivalents, and AsA in maize seedlings are presented in Figure 5. Cultivation of maize in soil contaminated with EMIMCl resulted in elevated concentrations of H_2_O_2_ and MDA equivalents, indicating enhanced oxidative stress. In addition, a slight initial decrease in endogenous AsA content relative to the control was observed, followed by an increase after exposure to the tested ionic liquid.

Exogenous application of AsA differentially affected the concentrations of H_2_O_2_, MDA equivalents, and AsA in maize seedlings, depending on the antioxidant dose. Only the 1 mM AsA treatment led to a slight reduction in H_2_O_2_ content. Both 1 and 2 mM AsA caused a modest decrease in MDA levels. Furthermore, supplementation with 1 and 2 mM AsA slightly increased endogenous AsA concentration compared with plants grown in EMIMCl-contaminated soil without antioxidant addition. In contrast, 0.5 mM AsA did not significantly influence any of the analyzed oxidative stress markers.

### 3.4. Multivariate Analysis of Physiological and Biochemical Responses of Maize Seedlings to EMIMCl Stress

**Figure 6:**
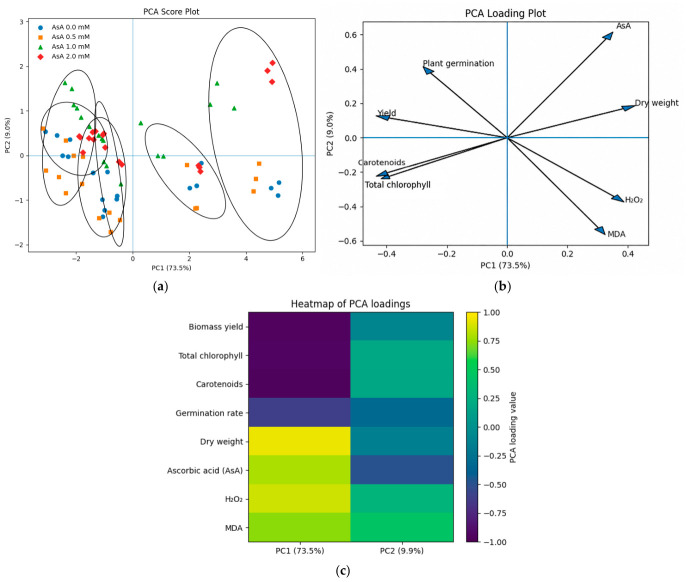
Principal component analysis (PCA) of physiological and biochemical responses of maize seedlings exposed to EMIMCl and treated with AsA. (**a**) Score plot showing sample distribution along PC1 and PC2; different point shapes indicate AsA concentrations, and ellipses represent 95% confidence intervals for EMIMCl treatments. (**b**) Loading plot illustrating relationships among the measured variables. (**c**) Heatmap of PCA loadings showing the contribution of individual variables to PC1 and PC2.

To integrate the results related to growth, photosynthetic performance, and oxidative stress in maize seedlings exposed to EMIMCl and exogenous AsA, principal component analysis (PCA) was performed. This approach enabled simultaneous evaluation of relationships among the measured parameters and identification of the main factors differentiating plant responses to chemical stress. The results are presented in Figure 6.

Principal component analysis (PCA) showed that the first two principal components captured 83.4% of the total variance in the dataset (PC1 = 73.5%, PC2 = 9.9%), indicating that the two-dimensional PCA model adequately reflected the relationships among the analyzed physiological and biochemical parameters. 

The score plot (Figure 6a) indicated that the main factor differentiating the samples was the PC1 axis. With increasing EMIMCl concentration, a gradual shift in the samples toward positive PC1 values was observed. Control samples and those exposed to low EMIMCl doses clustered mainly on the negative side of the axis, whereas treatments with the highest EMIMCl concentrations (500–1000 mg/kg of soil DW) formed a distinct cluster on the positive side. At the same time, the effect of AsA was less pronounced than that of EMIMCl alone—at higher AsA doses and intermediate EMIMCl concentrations (1–100 mg/kg of soil DW), partial overlap of the groups was observed, suggesting that exogenous application of AsA did not lead to complete separation of plant responses in the PCA space.

The loading plot (Figure 6b) revealed positive loadings of H_2_O_2_, MDA equivalents, AsA, and dry weight content along PC1, while growth- and photosynthesis-related parameters—including biomass yield, germination rate, and photosynthetic pigment content—were negatively associated with this component. The PCA loading heatmap (Figure 6c) confirmed the patterns observed in both the score and loading plots, supporting the consistency of the multivariate analysis.

## 4. Discussion

### 4.1. Effect of EMIMCl on the Growth and Development of Maize Seedlings

Ionic liquids (ILs) may influence this process in different ways. Depending on their chemical structure, ILs can reduce or completely inhibit seed germination—particularly at higher concentrations [33]—have no measurable effect [34], or even stimulate germination when applied at low doses [35]. Regardless of the direction of the effect, the magnitude of IL impact on germination and emergence is closely related to the applied concentration. Low doses often exert negligible or even stimulatory effects, whereas higher concentrations frequently suppress germination and reduce seedling emergence [33,34,35]. 

In the present study, more pronounced effects were observed in shoot and root elongation than in seedling emergence. The results demonstrate that EMIMCl negatively affects maize growth in a concentration-dependent manner. Only the lowest tested concentration (1 mg·kg^−1^ of soil DW) stimulated seedling growth and increased fresh biomass yield. Previous reports [36,37] suggest that low IL concentrations may promote plant growth, whereas higher levels can damage root cell membranes. Habibul et al. [38] reported that imidazolium-based ILs tend to accumulate predominantly in roots, although they may also be transported to stems and leaves. The extent of accumulation depends on both the applied concentration and the alkyl chain length of the IL.

Disturbances in root function and reduced root growth can lead to insufficient water and nutrient absorption. Consequently, decreased cellular turgor may occur, resulting in an increased proportion of dry weight in plant tissues. Elevated dry weight content is often interpreted as an indicator of impaired water balance, a common response to chemical and osmotic stress [39,40,41]. 

The results of the PCA further confirmed that increasing EMIMCl concentration was the main factor determining the response of maize. Samples exposed to the highest EMIMCl concentrations clustered separately from the control, indicating a strong relationship between stress intensity and growth inhibition as well as changes in physiological parameters.

### 4.2. Alterations in Photosynthetic Pigment Content and Chlorophyll Fluorescence in Maize Seedlings Exposed to EMIMCl

In the present study, a reduction in chlorophyll content was already apparent during visual assessment of the plants, as higher EMIMCl concentrations induced chlorotic symptoms on the leaves. These observations were subsequently confirmed by quantitative measurements of individual photosynthetic pigments.

Previous studies have similarly reported adverse effects of ionic liquids on pigment content in higher plants and algae [37,42,43]. Imidazolium-based ionic liquids may disrupt the lipid bilayer structure and impair chloroplast membrane integrity. Exposure to ILs can also promote excessive production of reactive oxygen species (ROS), which damage cellular membranes and thylakoid structures within chloroplasts. Chloroplast impairment may lead to leakage of chlorophyll, while the combined action of ILs and ROS may facilitate penetration into internal chloroplast compartments, further disturbing chlorophyll biosynthesis and photosynthetic machinery organization [39,42,44,45]. 

In addition to pigment concentration, chlorophyll fluorescence is considered a sensitive indicator of oxidative and photosynthetic stress. An increase in minimal fluorescence (F_0_) may reflect either reversible or irreversible inactivation of PSII or structural damage to thylakoid membranes. A reduction in variable fluorescence (F_v_) indicates decreased PSII efficiency. The ratios F_v_/F_m_ and F_v_/F_0_ provide information about the functional state of the PSII reaction center. Alterations in these parameters may result from EMIMCl-induced disturbances in the PSII electron transport chain or in the primary electron acceptor. A decline in F_v_/F_m_ is commonly interpreted as evidence of PSII reaction center damage. Such changes suggest the occurrence of photoinhibition under stress conditions, often accompanied by increased energy dissipation as heat and enhanced photodamage to the photosynthetic apparatus [37,42,46]. 

The PCA further revealed an opposite distribution of growth and photosynthetic parameters relative to oxidative stress markers, indicating that the deterioration of photosynthetic apparatus performance was closely associated with the intensification of the stress response induced by EMIMCl.

### 4.3. Effect of EMIMCl on Oxidative Stress in Maize Seedlings

The elevated H_2_O_2_ levels observed in maize seedlings cultivated in soil containing EMIMCl demonstrate that this ionic liquid induces oxidative stress in maize. Moreover, the magnitude of this effect increased with rising EMIMCl concentrations. Comparable increases in H_2_O_2_ content following exposure to ionic liquids have been reported by Zhang et al. [47] in duckweed treated with C_8_MIMBr, by Xu et al. [48] in wheat exposed to three C_8_MIM ionic liquids with different anions, and by Cvjetko Bubalo et al. [9] in barley seedlings subjected to four imidazolium-based ionic liquids.

MDA can Interact with functional groups in proteins, lipoproteins, and nucleic acids, lead to cellular injury. Disruption of membrane integrity may have serious physiological consequences for plants [47,48]. The increased MDA content detected in maize seedlings exposed to EMIMCl further confirms the occurrence of oxidative stress. As with H_2_O_2_, the intensity of these changes was positively correlated with the applied concentration of the ionic liquid. Similar detrimental effects of ionic liquids on crop species have been documented for barley [9], wheat [49,50], and rice [37]. 

According to the PCA, oxidative stress markers (H_2_O_2_ and MDA equivalents) showed a positive association with the PC1 axis, which corresponded to the EMIMCl concentration gradient. At the same time, growth and photosynthetic parameters were distributed in the opposite direction, confirming their negative relationship with stress intensity.

### 4.4. Role of Exogenous Ascorbic Acid in Alleviating EMIMCl-Induced Stress

Application of L-ascorbic acid (AsA) at low and moderate concentrations partially mitigated the adverse effects caused by EMIMCl. This protective response was reflected in improved plant growth, higher levels of photosynthetic pigments, and reduced accumulation of H_2_O_2_ and MDA. These findings indicate that AsA enhanced the antioxidant capacity of maize, thereby limiting oxidative damage.

Exogenous supplementation with L-ascorbic acid clearly attenuated the inhibitory effect of the ionic liquid on seedling emergence as well as shoot and root growth. The growth-promoting effect of AsA has also been documented in maize exposed to cadmium [18], wheat subjected to lead stress [51], rapeseed under drought conditions [52], peach trees experiencing water deficit [53], and wheat exposed to salinity [54]. The beneficial impact of AsA on plant development may result from stimulated synthesis of amino acids, proteins, and photosynthetic pigments. Moreover, AsA regulates cell division, differentiation, and senescence, and by strengthening antioxidant defenses, it protects lipids and proteins from oxidative injury [18,20,55,56,57,58]. 

External application of AsA—via seed priming, foliar spraying, or soil supplementation—leads to an increase in endogenous ascorbate content, which can positively modulate antioxidant metabolism in plants [59]. In the present study, maize seedlings grown in EMIMCl-contaminated soil exhibited a marked rise in AsA levels. This increase may represent a stress-induced response, as oxidative stress triggered by the ionic liquid likely stimulated the synthesis and accumulation of low-molecular-weight antioxidants, including ascorbate [60]. Under abiotic stress conditions, plants are known to upregulate genes encoding enzymes of the AsA biosynthetic pathway as well as those involved in the ascorbate–glutathione cycle [18]. Furthermore, membrane damage—indicated by elevated MDA equivalents content—activates defense mechanisms that may also contribute to increased AsA accumulation [60]. The effectiveness of exogenously applied AsA in reducing oxidative stress in maize seedlings was concentration-dependent. This observation is consistent with findings by Khazaei et al. [61] who demonstrated that acetylsalicylic acid applied at optimal concentrations enhanced drought tolerance in pepper (*Capsicum annuum* L.), and by Hassan et al. [62] who reported similar concentration-dependent protective effects of AsA against drought stress in barley (*Hordeum vulgare* L.).

The PCA results confirmed that EMIMCl was the main factor determining the physiological response of maize seedlings, which was reflected in the strong association of oxidative stress markers with positive PC1 values. The distribution of samples along the PC2 axis suggested an additional effect of exogenous AsA, although weaker than that of EMIMCl. The partial overlap of the groups indicates that AsA application did not lead to a complete reversal of the changes induced by EMIMCl and that the effectiveness of its action was dose-dependent.

### 4.5. Biphasic Effects of AsA Under Severe EMIMCl-Induced Stress

One of the key and novel findings of this study is the demonstration that the action of exogenous L-ascorbic acid under EMIMCl-induced stress follows a biphasic pattern and is strongly dependent on both its concentration and the severity of chemical stress. Low and moderate doses of AsA exerted a protective effect, whereas higher concentrations—particularly when combined with elevated levels of EMIMCl in the soil—not only failed to alleviate stress symptoms but markedly intensified them. This detrimental response was evidenced by a further increase in oxidative stress markers (H_2_O_2_ and MDA equivalents), along with deterioration of growth parameters and impairment of photosynthetic performance.

The dual nature of AsA activity is supported by numerous reports indicating that, despite its central role as an antioxidant, ascorbate may—under certain conditions—display pro-oxidative properties. AsA is a pivotal component of the plant redox network and functions within the ascorbate–glutathione cycle, where it modulates ROS levels and maintains cellular redox balance [59,63]. However, under intense stress conditions that overwhelm ROS-detoxifying systems, this balance may become disrupted.

At elevated concentrations, AsA can promote ROS generation through redox reactions, particularly in the presence of transition metal ions such as Fe^2+^ and Cu^+^, which catalyze Fenton-type reactions. In such circumstances, ascorbate may shift from acting solely as an ROS scavenger to facilitating the formation of highly reactive hydroxyl radicals, thereby enhancing lipid peroxidation and cellular damage [3,64]. This mechanism is especially relevant in plant cells exposed to xenobiotics that already compromise membrane integrity and organelle function.

Imidazolium-based ionic liquids have been shown to interact with biological membranes and proteins containing thiol groups, leading to disturbances in mitochondrial and chloroplast function and consequently to increased ROS production [9,13]. Under such conditions, the application of high doses of AsA may aggravate redox imbalance rather than counteract it. This explains the intensified oxidative stress symptoms observed in maize seedlings simultaneously exposed to high concentrations of EMIMCl and elevated levels of exogenous AsA in the present study.

The PCA results support this interpretation—at high concentrations of both EMIMCl and AsA, the samples shifted toward the region associated with oxidative stress markers, indicating that the effect of AsA was not universally protective.

## 5. Conclusions

In summary, exogenous AsA may attenuate oxidative damage in plants, at least partially restoring antioxidant balance through direct ROS scavenging and stimulation of antioxidant enzymes. However, the effectiveness of AsA in mitigating stress induced by ionic liquids depends on both the applied AsA concentration and the level of IL contamination. When IL exposure severely disrupts cellular function, exogenous AsA may exhibit limited protective capacity, and an improperly selected dose may further intensify oxidative stress rather than alleviate it. 

The obtained results demonstrated that the response of maize seedlings to the combined action of EMIMCl and exogenous AsA was dose-dependent. Low and moderate concentrations of AsA led to a partial mitigation of the negative effects of EMIMCl, as reflected by improved growth parameters, more favorable functioning of the photosynthetic apparatus, and reduced oxidative stress intensity. In contrast, high AsA concentrations, particularly under conditions of severe EMIMCl soil contamination, not only failed to exert a protective effect but also resulted in a further intensification of stress symptoms and deterioration of the analyzed physiological and biochemical parameters.

The obtained findings indicate that the effect of exogenous AsA is not universally protective and should be considered in the context of maintaining redox balance in plant cells. Disruption of this balance, especially under conditions of severe stress induced by ionic liquids, may lead to a shift of AsA from an antioxidant role toward a pro-oxidative mode of action. These observations highlight the complexity of plant response mechanisms to xenobiotic exposure and indicate that the effectiveness of exogenous stress regulators depends on the extent of cellular damage and the ability of plants to activate their own defense mechanisms.

Due to the lack of previous studies investigating the mitigation of ionic liquid phytotoxicity through the application of exogenous AsA, the present study represents one of the first reports demonstrating both the potential and the limitations of this strategy. The obtained results may contribute both to a better understanding of plant tolerance mechanisms in relation to chemical contaminants and to future environmental risk assessment associated with the presence of ILs in agroecosystems. At the same time, further studies are necessary to deepen understanding of this issue, including investigations across different plant species and expansion of the analysis to antioxidant enzyme activity, expression of genes involved in redox homeostasis regulation, evaluation of different AsA application methods, and determination of the long-term effects of IL exposure on plant growth and yield.

## Figures and Tables

**Table 1:** Shoot and root length of maize seedlings and percentage emergence of plants grown in soil contaminated with various concentrations of EMIMCl and supplemented with AsA at 0.5, 1, and 2 mM. Data are presented as means of 10 replicates (n = 10) ± standard deviation. Values within columns followed by the same letter do not differ significantly.

Concentration ILs [mg·kg^−1^ of Soil DW]	Concentration AsA [mM]	Root Length [cm]	Shoot Length [cm]	Number of Plants that Germinated
0 (control)	0	14.1 ± 1.5^abc^	24.8 ± 2.7^bcde^	9 ± 2^ab^
	0.5	15.0 ± 0.9^ab^	**29.2 ± 2.5^a^**	9 ± 1^ab^
	1	14.6 ± 1.1^abc^	26.3 ± 2.3^ab^	9 ± 0^ab^
	2	14.2 ± 1.2^abc^	24.0 ± 1.8^bcdef^	10 ± 1^ab^
1	0	14.6 ± 1.3^abc^	24.3 ± 2.3^bcde^	**10 ± 0^a^**
	0.5	**15.7 ± 1.2^a^**	25.7 ± 1.5^bcd^	10 ± 1^ab^
	1	15.1 ± 1.3^ab^	25.2 ± 2.7^bcde^	**10 ± 0^a^**
	2	14.6 ± 1.0^abc^	23.6 ± 2.2^bcdefg^	9 ± 0^ab^
50	0	13.5 ± 1.1^bcd^	22.2 ± 0.8^efg^	8 ± 1^bc^
	0.5	14.6 ± 1.3^abc^	26.1 ± 2.4^abc^	9 ± 2^ab^
	1	13.8 ± 1.1^bcd^	24.5 ± 2.7^bcde^	10 ± 1^ab^
	2	13.4 ± 1.1^bcd^	22.8 ± 1.8^cdefg^	10 ± 1^ab^
100	0	11.9 ± 1.3^d^	20.6 ± 1.8^fgh^	10 ± 1^ab^
	0.5	12.9 ± 1.4^cd^	22.4 ± 2.6^defg^	9 ± 1^ab^
	1	14.0 ± 1.1^abc^	25.1 ± 2.0^bcde^	**10 ± 0^a^**
	2	13.5 ± 0.9^bcd^	23.4 ± 2.5^bcdefg^	9 ± 1^ab^
500	0	7.4 ± 0.7^ef^	17.8 ± 2.2^h^	9 ± 1^ab^
	0.5	7.3 ± 1.1^efg^	17.8 ± 2.2^h^	9 ± 1^ab^
	1	9.1 ± 1.2^e^	24.5 ± 1.7^bcde^	8 ± 1^bc^
	2	7.8 ± 1.3^ef^	20.3 ± 2.2^gh^	9 ± 0^ab^
1000	0	6.4 ± 1.1^fg^	**11.1 ± 1.3^i^**	**6 ± 0^c^**
	0.5	6.6 ± 1.0^fg^	**10.7 ± 1.7^i^**	8 ± 1^bc^
	1	7.4 ± 0.9^ef^	**12.1 ± 1.2^i^**	9 ± 1^ab^
	2	**5.4 ± 1.1^g^**	**10.6 ± 1.5^i^**	8 ± 1^abc^

Bold values indicate the highest and lowest statistically distinct means within a given parameter.

**Table 2:** Fresh biomass and dry weight content of maize seedlings cultivated in soil contaminated with various concentrations of EMIMCl and supplemented with AsA at 0.5, 1, and 2 mM. Data are expressed as means of four replicates (n = 4) ± standard deviation. Values within columns marked with the same letter are not significantly different.

Concentration ILs [mg·kg^−1^ of Soil DW]	Concentration AsA [mM]	Fresh Biomass [g·pot^−1^]	Dry Weight Content [g·g^−1^ FW]
0 (control)	0	2.837 ± 0.281^bcd^	0.0993 ± 0.0028^g^
	0.5	3.330 ± 0.269^ab^	**0.0965 ± 0.0031^g^**
	1	3.184 ± 0.208^abc^	0.1018 ± 0.0014^fg^
	2	2.862 ± 0.322^bcd^	0.1027 ± 0.0005^fg^
1	0	3.111 ± 0.133^abc^	0.1007 ± 0.0026^fg^
	0.5	**3.498 ± 0.079^a^**	**0.0990 ± 0.0030^g^**
	1	3.171 ± 0.231^abc^	0.1008 ± 0.0020^fg^
	2	3.137 ± 0.335^abc^	0.1022 ± 0.0041^fg^
50	0	2.622 ± 0.107^cde^	**0.0986 ± 0.0010^g^**
	0.5	3.125 ± 0.133^abc^	**0.0988 ± 0.0016^g^**
	1	2.762 ± 0.185^bcd^	**0.0980 ± 0.0026^g^**
	2	2.649 ± 0.388^cd^	**0.0987 ± 0.0021^g^**
100	0	2.058 ± 0.054^efg^	**0.0979 ± 0.0008^g^**
	0.5	2.339 ± 0.176^def^	**0.0999 ± 0.0017^g^**
	1	2.807 ± 0.006^bcd^	**0.0995 ± 0.0005^g^**
	2	2.707 ± 0.053^bcd^	**0.0983 ± 0.0029^g^**
500	0	1.474 ± 0.004^hi^	0.1157 ± 0.0050^cde^
	0.5	1.438 ± 0.063^hi^	0.1171 ± 0.0020^bcd^
	1	1.997 ± 0.045^fgh^	0.1082 ± 0.0014^ef^
	2	1.592 ± 0.147^ghi^	0.1134 ± 0.0015^de^
1000	0	**1.078 ± 0.076^i^**	**0.1254 ± 0.0025^a^**
	0.5	**1.127 ± 0.013^i^**	0.1214 ± 0.0012^abc^
	1	1.608 ± 0.111^ghi^	0.1223 ± 0.0026^abc^
	2	**1.041 ± 0.143^i^**	0.1242 ± 0.0040^ab^

Bold values indicate the highest and lowest statistically distinct means within a given parameter.

**Table 3:** Chlorophyll fluorescence parameters in maize seedlings cultivated in soil contaminated with various concentrations of EMIMCl and supplemented with AsA at 0.5, 1, and 2 mM. Data are expressed as means of four replicates (n = 4) ± standard deviation. Values within columns followed by the same letter do not differ significantly.

Concentration ILs [mg·kg^−1^ of Soil DW]	Concentration AsA [mM]	F_0_	F_m_	F_v_	F_v_/F_m_	F_v_/F_0_
0 (control)	0	**199 ± 4^e^**	970 ± 40^bc^	771 ± 38^a^	0.794 ± 0.008^ab^	3.869 ± 0.170^a^
	0.5	**214 ± 26^e^**	938 ± 37^cd^	725 ± 34^a^	0.772 ± 0.024^abc^	3.431 ± 0.434^ab^
	1	219 ± 20^de^	1025 ± 42^abc^	**819 ± 82^a^**	**0.797 ± 0.049^a^**	3.732 ± 0.126^a^
	2	**201 ± 5^e^**	989 ± 27^abc^	788 ± 26^a^	0.796 ± 0.007^ab^	**3.915 ± 0.153^a^**
1	0	**202 ± 4^e^**	988 ± 39^abc^	786 ± 37^a^	0.795 ± 0.007^ab^	3.892 ± 0.153^a^
	0.5	**204 ± 5^e^**	994 ± 25^abc^	790 ± 22^a^	0.795 ± 0.005^ab^	3.884 ± 0.101^a^
	1	**207 ± 12^e^**	982 ± 44^abc^	774 ± 33^a^	0.788 ± 0.005^ab^	3.739 ± 0.116^a^
	2	**200 ± 11^e^**	965 ± 70^bcd^	764 ± 60^a^	0.792 ± 0.007^ab^	3.813 ± 0.159^a^
50	0	**200 ± 5^e^**	980 ± 30^abc^	780 ± 25^a^	0.796 ± 0.003^ab^	3.903 ± 0.068^ga^
	0.5	**198 ± 18^e^**	969 ± 60^bc^	756 ± 95^a^	0.778 ± 0.055^abc^	3.808 ± 0.215^a^
	1	**202 ± 5^e^**	969 ± 55^bc^	767 ± 53^a^	0.791 ± 0.010^ab^	3.805 ± 0.223^a^
	2	**213 ± 13^e^**	982 ± 34^abc^	769 ± 36^a^	0.783 ± 0.014^abc^	3.624 ± 0.298^a^
100	0	**211 ± 3^e^**	1014 ± 22^abc^	803 ± 21^a^	0.791 ± 0.005^ab^	3.807 ± 0.103^a^
	0.5	**206 ± 15^e^**	953 ± 51^abc^	747 ± 60^a^	0.782 ± 0.025^abc^	3.646 ± 0.487^a^
	1	**210 ± 3^e^**	1004 ± 28^abc^	794 ± 28^a^	0.790 ± 0.006^ab^	3.787 ± 0.136^a^
	2	**209 ± 7^e^**	995 ± 51^abc^	786 ± 45^a^	0.789 ± 0.006^ab^	3.752 ± 0.130^a^
500	0	325 ± 58^c^	1095 ± 65^ab^	772 ± 79^a^	0.705 ± 0.052^abc^	2.473 ± 0.633^c^
	0.5	312 ± 50^cd^	**1121 ± 71^a^**	809 ± 42^a^	0.722 ± 0.033^abc^	2.648 ± 0.492^bc^
	1	327 ± 64^c^	1036 ± 40^abc^	709 ± 68^a^	0.686 ± 0.082^abc^	2.262 ± 0.609^c^
	2	350 ± 80^c^	1056 ± 85^abc^	705 ± 58^a^	0.673 ± 0.095^c^	2.117 ± 0.618^c^
1000	0	315 ± 59^cd^	1078 ± 78^abc^	763 ± 47^a^	0.709 ± 0.040^abc^	2.490 ± 0.545^c^
	0.5	328 ± 59^c^	1043 ± 44^abc^	715 ± 63^a^	0.686 ± 0.055^bc^	2.249 ± 0.533^c^
	1	**679 ± 61^a^**	1062 ± 90^abc^	**383 ± 55^b^**	**0.311 ± 0.087^d^**	**0.503 ± 0.195^d^**
	2	568 ± 43^b^	**819 ± 92^d^**	**252 ± 66^b^**	**0.304 ± 0.047^d^**	**0.496 ± 0.151^d^**

Bold values indicate the highest and lowest statistically distinct means within a given parameter.

## Data Availability

The original contributions presented in this study are included in the article/Supplementary Material. Further inquiries can be directed to the corresponding author.

## Supplementary Materials

The following supporting information can be downloaded at: https://www.mdpi.com/article/10.3390/toxics14070589/s1, Table S1: Results of two-way ANOVA showing the effects of concentration ionic liquids (IL), ascorbic acid (AsA), and their interaction (IL × AsA) on the analyzed growth, physiological, and biochemical parameters.
