# Supplementary material for: The Effect of 1-Ethyl-3-Methylimidazolium Chloride on Oxidative Stress and the Functioning of the Photosynthetic Apparatus in Maize Seedlings—The Modulatory Role of Exogenous Ascorbic Acid
Source: Toxics. 2026 Jul 3;14(7):589. doi: 10.3390/toxics14070589 (PMC13417908; doi:10.3390/toxics14070589)
Supplement: Supplementary file 1 [file toxics-14-00589-s001.zip › toxics-4376491-supplementary.pdf]

Supplementary Table S1. Results of two-way ANOVA showing the effects of concentration ionic liquids (IL), ascorbic acid (AsA), and their interaction (IL × AsA) on the analyzed growth, physiological, and biochemical parameters.

Parameter	IL (F)	IL (p)	AsA (F)	AsA (p)	IL × AsA (F)	IL × AsA (p)
Fresh biomass	235.00	<0.001	15.39	0.000	4.28	<0.001
Dry weight	206.80	<0.001	1.40	0.253	2.60	0.006
Number of plants that germinated	12.22	0.000	3.68	0.018	3.20	0.002
H_2O_2	385.60	<0.001	140.80	<0.001	21.30	<0.001
MDA	62.01	<0.001	16.74	<0.001	9.33	<0.001
AsA	207.45	<0.001	34.79	<0.001	7.13	<0.001
Chl a	5082	<0.001	6643	<0.001	671	<0.001
Chl b	32718	<0.001	6871	<0.001	756	<0.001
Chl (a+b)	46545	<0.001	7354	<0.001	685	<0.001
Car	61659	<0.001	5410	<0.001	741	<0.001
Chl a/Chl b	4604	<0.001	2720	<0.001	1313	<0.001
Chl(a+b)/Car	5082	<0.001	631	<0.001	521	<0.001
F_0	150.27	<0.001	20.21	<0.001	16.24	<0.001
F_m	7.76	<0.001	4.44	0.006	4.06	<0.001
F_v	57.25	<0.010	18.55	<0.001	15.74	<0.001
F_v/F_m	124.31	<0.001	22.14	<0.001	19.43	<0.001
F_v/F_0	139.31	<0.001	10.52	<0.001	6.88	<0.001
Shoot lenght	402.80	<0.001	9.70	<0.001	2.43	0.003
Root lenght	270.32	<0.001	21.48	<0.001	6.23	<0.001
